# 

**Published:** 1909-05

**Authors:** 


### BOOKS RECEIVED.

Textbook of Embryology, by Frederick Randolph Bailey, A.M., M.D., adjunct professor of histology and embryology, college of physicians and surgeons (Medical Department of Columbia University), and Adam Marion Miller, A.M., instructor in histology and embryology at the same institution. With 515 illustrations. New York: William Wood & Company. 1909.

Elementary Practical Treatise on Diseases of the Larynx and Pharynx by Dr. E. J. Moure, surgeon in charge of the nose, ear and throat department of the Faculty of Medicine, Bordeaux. Translated and adapted by J. Malcom Farquharson, M.B. F.R.C.R., Edin., lecturer on diseases of nose, ear and throat in the School of Medicine of the Royal Colleges, Edinburgh, etc., with 210 illustrations. New York: Rebman Company. 1909.

Conservative Gynecology and Electro Therapeutics: A Practical Treatise on the Diseases of Women and Their Treatment by Electricity by G. Betton Massey, M.D., attending surgeon to the American Oncologic Hospital, Philadelphia; Fellow and Ex-President of the American Electro-Therapeutic Association; Member of the American Medical Association, etc. Sixth edition, thoroughly revised. Royal octavo, 462 pages. Illustrated with twelve (12) original, full-page, chromo-lithographic plates and fifteen (15) full-page half-tone plates of photographs taken from nature, and numerous engravings in the text. Bound in extra cloth. Price $4.00 net. F. A. Davis Company, publishers, 1914-16 Cherry Street, Philadelphia, Pa.

Annual Report of the Surgeon General of the Public Health and Marine Hospital Service of the United States, giving the operations of the service, fourth fiscal year, 1908. Washington Government Printing Office. 1909.

Mortality Statistics, Department of Commerce and Labor, Bureau of the Census, 1907 Eighth Annual Report, by S. N. D. North, Director. Washington: Government Printing Office. 1909.

Atlas and Epitome of External Diseases of the Eye. By Professor Dr. O. Haab, of Zurich. Edited, with additions, by George E. deSchweinitz, M.D., Professor of Ophthalmology, University of Pennsylvania. Third revised edition. With 101 colored lithographic illustrations on 46 plates and 244 pages of text. Philadelphia and London: W. B. Saunders Company, 1909. Cloth, $3.00 net.

Atlas and Epitome of Ophthalmoscopy and Ophthalmoscopic Diagnosis. By Professor Dr. O. Haab, of Zurich. Edited, with additions, by George E. deSchweinitz, M.D., Professor of Ophthalmology, University of Pennsylvania. Second revised edition. With 152 colored lithographic illustrations and 94 pages of text. Philadelphia and London: W. B. Saunders Company, 1909. $3.00 net.

